# Improved Energy Storage Performance of Composite Films Based on Linear/Ferroelectric Polarization Characteristics

**DOI:** 10.3390/polym16081058

**Published:** 2024-04-11

**Authors:** Chen Chen, Lifang Shen, Guang Liu, Yang Cui, Shubin Yan

**Affiliations:** 1 Nanxun Innovation Institute, Zhejiang University of Water Resources and Electric Power, Hangzhou 310018, China; cuiy@zjweu.edu.cn; 2 School of Electrical Engineering, Zhejiang University of Water Resources and Electric Power, Hangzhou 310018, China; shenlf@zjweu.edu.cn (L.S.); yanshb@zjweu.edu.cn (S.Y.)

**Keywords:** polarization characteristics, PVTC/BOPP bilayer films, interfaces, energy storage properties

## Abstract

The development and integration of high-performance electronic devices are critical in advancing energy storage with dielectric capacitors. Poly(vinylidene fluoride-trifluoroethylene-chlorofluoroethylene) (PVTC), as an energy storage polymer, exhibits high-intensity polarization in low electric strength fields. However, a hysteresis effect can result in significant residual polarization, leading to a severe energy loss, which impacts the resultant energy storage density and charge/discharge efficiency. In order to modify the polarization properties of the polymer, a biaxially oriented polypropylene (BOPP) film with linear characteristics has been selected as an insulating layer and combined with the PVTC ferroelectric polarization layer to construct PVTC/BOPP bilayer films. The hetero-structure and polarization characteristics of the bilayer film have been systematically studied. Adjusting the BOPP volume content to 67% resulted in a discharge energy density of 10.1 J/cm^3^ and an energy storage efficiency of 80.9%. The results of this study have established the mechanism for a composite structure regulation of macroscopic energy storage performance. These findings can provide a basis for the effective application of ferroelectric polymer-based composites in dielectric energy storage.

## 1. Introduction

Dielectric capacitors exhibit high power density and fast charge/discharge rates [1,2] and are widely used in pulse power devices in electrical/electronic engineering [3]. When compared with inorganic dielectric capacitors, polymer-based systems offer distinct advantages in terms of high breakdown strength, low dielectric loss, and low density. Moreover, the associated flexibility and processability offer many options for the micro-structure design and macroscopic performance control of thin film capacitors and embedded capacitors. Polymer-based dielectric capacitors have significant scope for application in high-energy weapons, electronic communication, and new energy vehicles [4,5]. The energy storage density of pure polymers is generally low and cannot meet the practical requirements of many compact pulse power devices. Current research in engineering dielectrics has focused on improvements in the energy storage characteristics of polymers. The latter depends on the polymer breakdown and polarization characteristics and the interrelationship of these factors. Improvements in the energy storage density of polymers require the targeted development of polymer-based composite systems [6].

Doping polymers with high dielectric constant inorganic fillers is the most common modification strategy used to enhance the energy storage performance of the composite due to the dielectric properties and interface polarization effect of the fillers. Tang et al. increased the relative dielectric constant of the composite to 17.5 at room temperature (ca. 25 °C) by doping a polyvinylidene fluoride (PVDF) matrix with 7.5 vol.% Ba_0.2_Sr_0.8_TiO_3_ paraelectric nanofibers. When an electric field of 450 kV/mm was applied, the discharge energy density reached 14.86 J/cm^3^, which was 42.9% higher than pure PVDF. However, a hysteresis effect due to polarization/depolarization resulted in a ca. 40% energy loss and a resultant energy storage efficiency of 60% [7]. Zhang and co-workers enhanced the interfacial polarization effect significantly by doping PVDF with BaTiO_3_@TiO_2_ fibers, generating a maximum polarization intensity of 9 μC/cm^2^. The application of a 650 kV/mm electric field delivered a discharge energy density of 21.2 J/cm^3^, where the energy loss was 25% [8]. The introduction of high dielectric fillers can improve the dielectric constant of the polymer to a certain extent, but it ultimately leads to a decrease in breakdown strength. This may be attributed to the large dielectric difference between the polymer matrix and inorganic fillers in addition to the local electric field distortion caused by interface incompatibility. An accumulation of ceramic filler aggregates results in conductive pathways along the electric field direction in the polymer matrix, causing a decrease in the breakdown strength of the composite material and an inability to achieve a high-energy storage density.

Doping the polymer matrix with the varying content, type, and morphology of the functional filler can achieve a high breakdown, high polarization, and a high thermal conductivity layer. By utilizing the dielectric mismatch between adjacent film layers to regulate the spatial electric field distribution, an interface barrier effect is formed that effectively blocks the breakdown path and improves the energy storage performance of the composite film. Introducing nanoscale fillers into the polymer generates a hard layer and soft layer, representing film layers with high breakdown strength and high polarization strength, respectively. The hard layer helps to suppress the formation of conductive channels, while the soft layer provides a high polarization intensity in high-electric-strength fields [9,10]. The combination of different hard and soft layers and the interfacial potential barriers between the film layers have a significant impact on the dielectric properties of composite films. The soft layer typically involves barium titanate (BaTiO_3_) and titanium dioxide (TiO_2_) doping [11,12], whereas high-insulation fillers (two-dimensional nano sheets) are associated with hard layers [13,14]. Research has shown that the formation of a “hard–soft” bilayer composite film can effectively reduce the leakage current density and improve overall dielectric properties. Liu et al. incorporated BaTiO_3_ particles into poly(vinylidene fluoride-co-hexafluoropropene) (P(VDF-HFP)) and constructed a bilayer composite film with poly(tetrafluoroethylene vinylidene fluoride hexafluoropropene) (THV). At a field strength of 575.6 kV/mm, a discharge energy density (*U*_d_) of 22.7 J/cm^3^ and a discharge efficiency (*η*) of 79% were obtained [15]. In addition to the hard–soft double-layer structure, sandwich composite films with a “soft–hard–soft” arrangement have also been studied. Li et al. utilized 2% MgO nw/P(VDF-HFP) as the hard layer and 20% BaTiO_3_ nw/P(VDF-HFP) as the soft layer [16]; at an applied field strength of 416 MV/m, the *U*_d_ was 15.5 J/cm^3^. While the soft–hard–soft structure can increase the energy storage density, there are certain negative effects. Choosing a nano-filler polarization layer as the outer layer facilitates electrode charge injection under high-electric fields, and the consequent improvement of energy storage is limited.

In multi-layer polymer-based composite films, the polarization effect at the interlayer interface perpendicular to the direction of the applied electric field is beneficial for improving dielectric properties. The inorganic filler and polymer film are combined to form a multi-layer composite film structure. As the number of film layers increases, the interlayer interface component increases, hindering the expansion of the electrical tree along the direction of the electric field. Jiang et al. investigated the influence of the number of layers on the dielectric properties, preparing multi-layer composite films by alternately stacking P(VDF-HFP) and BT/P(VDF-HFP) composite fiber layers [16]. As the number of interlayer interfaces increased, the dielectric constant of the multi-layer structure showed a slight increase, while the dielectric loss gradually decreased. The application of the phase field simulation also established that the interlayer interface suppressed the movement of charge carriers, reducing the leakage current density and improving overall energy storage performance. Differences in the relative dielectric constant and conductivity of adjacent film layers in the alternating multi-layer thin film result in a severe distortion of the electric field at the interface. Gradient structured composite films can regulate the distribution of electric field gradients by continuously changing the electrical parameters. Taking a three-layer gradient structure thin film as an example, the relative dielectric constant of each film layer gradually increases from top to bottom. Under an external electric field, the electric field distributed by each film layer gradually decreases. Assuming that the electrical breakdown is caused by the migration of charge carriers from electrode A to electrode B, the electrical tree will grow downwards from the upper film with a higher electric field strength. When the electrical tree grows to reach interface A, the electric field strength of the middle film layer is lower than the upper layer, which limits the growth of the electrical tree to a certain extent. A minor component of electrical trees will continue to grow in the middle film layer. When the electrical tree grows to interface B, the lower film bears a lower electric field strength, and it is possible to terminate the growth of the electrical tree. Assuming that electrical breakdown is caused by the migration of charge carriers from electrode B to electrode A, the electric field strength of the lower film is lower, and it is difficult to induce electrical tree branches. The penetration of electrical tree branches through the lower film requires a higher electric field strength to improve the overall breakdown field strength of the composite film [17].

Multi-layer nano-composite films not only integrate the high dielectric constant of inorganic nano-fillers and the high breakdown strength of polymer films, but the multi-layer structure also enables an adjustment of filler content, circumventing filler aggregation with a consequent decline in performance caused by high doping content. Due to the difference in the dielectric constant between film layers in nanoscale multi-layer structures, the spatial electric field is redistributed, which can effectively suppress the development of electrical tree branches and improve the dielectric properties. The introduction of nano-fillers not only serves to optimize polymer energy storage performance but also leads to an electric field distortion near the filler due to the large dielectric differential between the filler and matrix, which is not conducive to improved energy storage performance. Some polymers require biaxial stretching treatment during production, where the incorporation of fillers can generate many defects during the stretching process that inhibit flexibility. The complexity of the preparation process limits the feasibility of large-scale production. The utilization of linear polymers with good insulation properties as the insulation layer and ferroelectric polymers, as the polarization layer enables the construction of a fully organic multi-layer thin film by stacking growth. Adjusting the volume ratio or stacking method of the insulation and polarization layers facilitates the formation of a composite thin film that combines high-energy density and high discharge efficiency. As a new generation of high-energy density dielectrics, PVDF-derived copolymers exhibit high discharge energy density, but the discharge efficiency sharply decreases (<70%) under a high field strength. Researchers have employed PVDF as the I-layer and P(VDF-TrFE-CTFE) as the P-layer. Due to the difference in the dielectric constant between the film layers, the middle P-layer bears a lower electric field and is less prone to premature breakdown, which enhances the breakdown field strength of the composite film. When a field strength of 660 MV/m was applied, *U*_d_ reached 20.86 J/cm^3^, with a *η* of 62%. The use of polymers such as polyimide (PI) and polymethyl methacrylate (PMMA) with high breakdown strength and high discharge efficiency (>90%) as the I-layer to construct linear/ferroelectric hetero-structure composite films represents an effective strategy for achieving a high density and high rate [18,19].

In this study, we have set out to address PVTC polymer polarization, preparing a bilayer film with a linear/ferroelectric hetero-structure by incorporating BOPP. The difference in the dielectric properties of the linear and ferroelectric polymers is utilized to redistribute the electric field within the bilayer film. At a BOPP volume content of 67%, the PVTC/BOPP bilayer film exhibited excellent energy storage characteristics. At an electric field strength of 550 kV/mm, the energy storage density and charge/discharge efficiency reached 10.1 J/cm^3^ and 80.9%, respectively. The organic multi-layer composite structure utilizes the performance characteristics of the constituent polymers, avoiding mismatches between inorganic fillers and polymer matrices, and simplifying materials preparation. Moreover, the hetero-structure interface enhances the dielectric properties of the multi-layer composite film. When compared with nano-composite multi-layer structures, all organic multi-layer films have shown significant advantages in terms of charge/discharge cycle stability and mechanical bending.

## 2. Experimental Section

### 2.1. Raw Materials

BOPP films with different thicknesses (5 μm, 7.5 μm, 10 μm, and 15 μm) and poly(vinylidene fluoride-trifluoroethylene-chlorofluoroethylene) (PVTC) powder were purchased from PolyK Technologies (Philipsburg, PA, USA); the ratios of vinylidene fluoride (VDF), trifluoroethylene (TrFE), and chlorofluoroethylene (CFE) monomers were 62%, 31%, and 7%, respectively. The N,N-dimethylformamide (DMF) reagent was obtained from the Sinopharm Chemical Reagent Co., Ltd., Shanghai, China.

### 2.2. Preparation of the Bilayer Films

BOPP films of different thicknesses were placed on a glass substrate for fixation. A PVTC/DMF solution was then cast on the BOPP film after vacuum degassing. A bilayer wet organic film was obtained in a stepwise thermal treatment from 80 °C to 200 °C in a vacuum oven. The sample was then immediately quenched in ice water and dried to give the required bilayer films. The volume ratios of BOPP in the bilayer films were 33%, 50%, and 67%.

### 2.3. Structural Characterization and Performance Testing

The crystal structure of the polymer film was determined using a PANalytical Empire (PANARKO, Alemlo, The Netherlands) unit at an operating voltage of 40 kV and 40 mA current. Characterization of the molecular structure and chemical bonding in the polymer films was performed using JASCO 6100 equipment (EQUINOX55 from Bruker, Karlsruhe, Germany). The film morphology and structure were assessed using FESEM and Hitachi SU8020 UHR equipment (Hitachi, Tokyo, Japan). The energy level structure and bandgap widths of the polymer films were obtained with ultraviolet and visible spectrophotometry (UV-Vis) (UV-3600i Plus from Shimadzu, Kyoto, Japan) the lowest and highest energy levels were determined with ultraviolet photoelectron spectroscopy (UPS) (Thermo Fischer, ESCALAB 250Xi, Waltham, MA, USA). The dielectric properties (dielectric constant and dielectric loss) of the polymer films under different frequency conditions were assessed using a broadband impedance analyzer (GmbH Novo control Alpha-A, Montabaur, Germany) at room temperature; the sample was 30 mm in length and 30 mm in width. The DC breakdown characteristics of the composite films were tested using the DC breakdown module of the dielectric ferroelectric integrated test system (PolyK Technologies, Philipsburg, PA, USA), with a boosting rate of 200 V/s.The polarization strength was evaluated using the Radiant Premier II (Albuquerque, NM, USA) ferroelectric testing system to generate polarization curves at different electric field strengths. The energy storage parameters (discharge energy density and charge/discharge efficiency) were obtained from an integration of the polarization curves.

## 3. Results and Discussion

**Figure 1:**
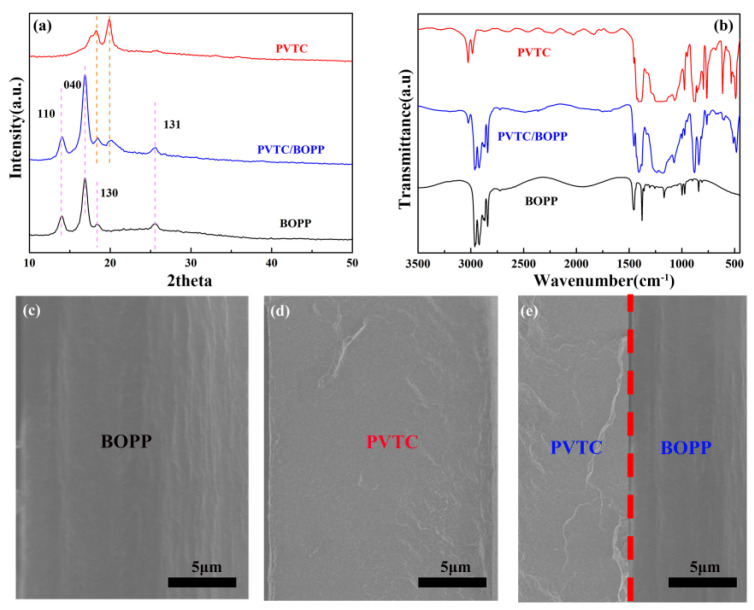
Micro-structure analyses of polymer films. (**a**) XRD patterns. (**b**) FTIR spectra. (**c**–**e**) SEM images.

In order to establish the successful preparation of bilayer thin films, XRD and FTIR techniques were employed to analyze the phase composition of the composite films, and scanning electron microscopy was used to assess the microstructure. As shown in Figure 1a, the diffraction peaks at 17.9°, 18.5°, and 20.1° correspond to PVTC [20,21,22], and the peaks at 14.04°, 16.85°, and 18.48° may be attributed to BOPP [23,24]. These characteristic peaks were observed in both pristine films and the prepared bilayer films. The FTIR spectra of the polymer films are presented in Figure 1b, where the peaks at 976 cm^−1^, 812 cm^−1^, 532 cm^−1^, and 776 cm^−1^ are due to the α- and γ-phases of PVTC [25,26,27]. The absorption peak at 1720 cm^−1^ may be attributed to (C=O) stretching, which is the result of oxidative degradation [28,29,30]. All the characteristic peaks were present in the bilayer films. The cross-sectional SEM images are given in Figure 1c–e, where the thickness of composite films is ca. 15 μm; there is no clearly discernible interface separation in the bilayer films.

**Figure 2:**
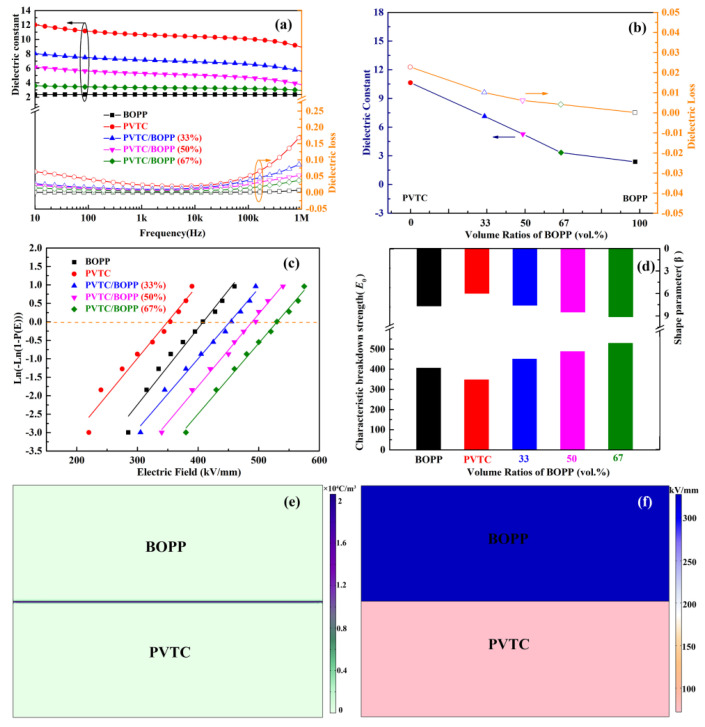
(**a**) Frequency-dependent changes of the dielectric constant and dielectric loss of the bilayer films. (**b**) The relationship between dielectric properties and BOPP volume ratios in bilayer films measured at 1 kHz. (**c**) Weibull distribution of the bilayer films. (**d**) Characteristic breakdown strength (*E*_b_) and shape parameter (*β*). (**e**) The space charge density simulation for the bilayer film. (**f**) The distribution of electric field simulated for the bilayer film.

The first key factor in determining energy storage performance is the dielectric property of the polymer films. The dielectric constants (Figure 2a) of the synthesized PVTC/BOPP bilayer films lie between the dielectric constants of pristine BOPP (ca. 2.37 at 1 kHz) and pristine PVTC (ca. 10.64 at 1 kHz). As shown in Figure 2b, the bilayer dielectric constants films at a frequency of 1 kHz are 7.14, 5.27, and 3.33, respectively. The dielectric constants decreased with increasing BOPP volume fraction in accordance with the theoretical dielectric constant law [31,32,33]. The dielectric loss associated with the PVTC/BOPP bilayer film is lower than that of the PVTC film in a given frequency range, which results from the lower dielectric loss of the incorporated BOPP film with linear polarization characteristics. As shown in Figure 2b, the dielectric loss of the PVTC/BOPP bilayer films at a frequency of 1 kHz is 0.010, 0.006, and 0.004, respectively. This dielectric loss includes polarization loss and conductivity loss. The dielectric loss of the bilayer films decreases with increasing frequency in the low frequency range but gradually increases in the high frequency range. This response demonstrates the relaxation characteristics of these dielectric materials, notably Maxwell–Wagner (M-W) relaxation at low frequencies and the α-relaxation phenomenon at high frequencies. In order to further investigate the factors that influence energy storage, the breakdown performance of the bilayer film was tested. Following a linear fit of the data, the Weibull distribution curve was generated [34,35], as shown in Figure 2c. The data analysis has revealed that the introduction of linear polymers results in an increase in the breakdown field strength (*E*_b_) of the PVTC/BOPP bilayer film relative to the pristine PVTC film (ca. 350 kV/mm). When the breakdown probability is 63.2%, the breakdown field strengths (*E*_b_) of the PVTC/BOPP bilayer films are 450.8 kV/mm, 487.9 kV/mm, and 528.9 kV/mm, respectively; the associated slopes of the breakdown data (*β*) are 7.6, 8.5, and 9.1. The larger the *E*_b_ value, the higher the fitting slope of the breakdown data is. The increase in breakdown field strength means that the dielectric difference between linear BOPP and ferroelectric PVTC results in a redistribution of the electric field. The BOPP layer with a smaller dielectric constant distributes a higher local electric field, and the breakdown field strength borne by PVTC in the bilayer film decreases. Simulation using the method in COMSOL Multiphysics (Figure 2e) has established that space charges tend to accumulate at the interface between PVTC and BOPP layers in the bilayer films due to the large mismatch in the electrical resistivity or dielectric constant of adjacent layers. The interfacial charges give rise to an increase in the average dielectric constant of the bilayer films [36,37]. The data presented in Figure 2f illustrate that the introduction of a linear layer is accompanied by a redistribution of the electric field inside the bilayer film.

**Figure 3:**
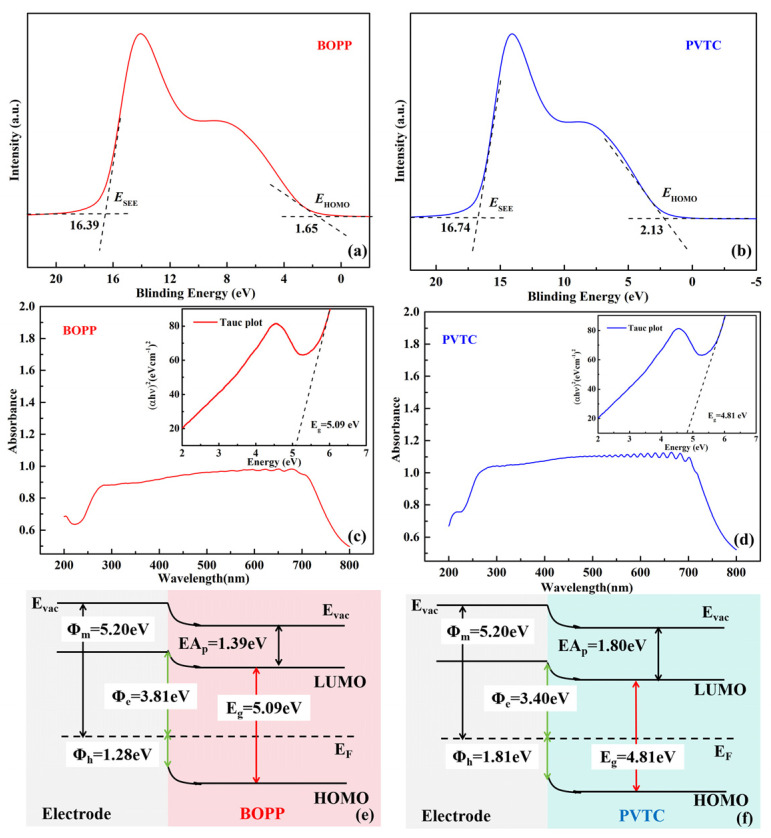
UPS spectra of (**a**) single-layer BOPP films and (**b**) single-layer PVTC films. UV-Vis absorption spectra (with inset showing the *T*_auc_ analyses) of (**c**) single-layer BOPP films and (**d**) single-layer PVTC films. Energy band structure diagram of (**e**) Au/BOPP and (**f**) Au/PVTC.

In order to gain a better understanding of the performance mechanism, the energy band structures of the PVTC ferroelectric and BOPP linear layers were analyzed. The UPS spectra of the polymer films are presented in Figure 3a,b, where the minimum binding energy (*E*_HOMO_) associated with the right-hand side and the maximum binding energy (*E*_SEE_) in the left-hand side can be obtained and the ionization potential (*IP*) calculated using Equation (1).

(1) $$IP_{HOMO}=h\nu -(E_{SEE}-E_{HOMO})$$

The calculated *IP*_HOMO_ allows for a determination of the positions of HOMO energy levels in the inorganic layer and polymer thin film; the positions of LUMO energy levels can be obtained from the bandgap width. Given that the work function of Au is 5.2 eV, combined with the HOMO and LUMO energy levels, the electron (*ϕ*_e_) and hole(*ϕ*_h_) potential barriers for each material can be calculated [38,39].

The UV-Vis analysis of the PVTC and BOPP films was undertaken to calculate the bandgap widths, as shown in Figure 3c,d. The UV absorption intensity of the material is presented as a function of the photoelectron wavelength. The relationship between the absorption rate and photon energy in the bandgap can be expressed by *T*_auc_ Equation (2):

(2) $${\left(\alpha h\nu \right)}^{2}=B\left(h\nu -E_{g}\right)$$

where *α* represents the absorption coefficient, *hν* is the photon energy, *h* is the Planck constant (4.1356710^−15^ eV•s), ν is the frequency of incident photons (*ν* = *c*/*λ*, where *c* is the speed of light (3 × 10^8^ m/s) and *λ* is the wavelength of the incident light), *B* is the proportional constant, and *E*_g_ is the bandgap width of the material. The calculated *E*_g_ values for BOPP and PVTC are 5.09 eV and 4.81 eV, respectively [40,41]. Based on the calculated bandgap width, electron barrier height, and hole barrier height, the band structures were generated and are shown in Figure 3e,f. The barrier height associated with hole and electron injection in the case of BOPP and PVTC are 3.81 eV, 3.40 eV and 1.28 eV, and 1.80 eV, respectively. Given the variation of the electron barrier height at the electrode dielectric interface, the introduction of a higher insulation layer can suppress the charge injection at the interface between the electrode and dielectric, resulting in an improved breakdown resistance for the bilayer films [42,43,44,45].

**Figure 4:**
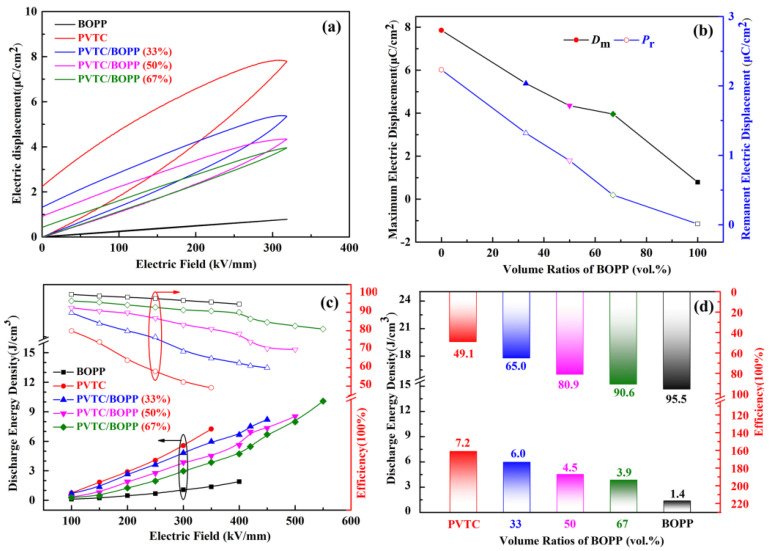
(**a**) The polarization characteristics of PVTC/BOPP bilayer films at the same electric field strength. (**b**) Maximum electric displacements (*D*_m_) and Remanent electric displacements (*P*_r_) of PVTC/BOPP bilayer films at the same electric field strength. (**c**) Relationship between the applied electric field and energy storage performances of PVTC/BOPP bilayer films. (**d**) Relationship between BOPP volume ratios and energy storage performances at the same electric field strength.

The incorporation of linear BOPP in constructing bilayer films serves to improve energy storage performance to a certain extent. In order to determine the relevant energy storage parameters, the charge/discharge curves of the polymer film were measured and are shown in Figure 4a,b. It can be seen that at the same electric field intensity, the PVTC/BOPP bilayer film exhibits a smaller curve area relative to the PVTC film. The maximum electric displacements (*D*_m_) at the same electric field intensity for the PVTC/BOPP(33%), PVTC/BOPP(50%), and PVTC/BOPP(67%) bilayer films are 5.38 μC/cm^2^, 4.35 μC/cm^2^, and 3.96 μC/cm^2^, respectively. The remanent electric displacements (*P*_r_) at the same electric field intensity for PVTC/BOPP(33%), PVTC/BOPP(50%), and PVTC/BOPP(67%) bilayer films are 1.32 μC/cm^2^, 0.92 μC/cm^2^, and 0.43 μC/cm^2^, respectively. These results indicate that BOPP, a linear dielectric with a smaller dielectric constant, can improve to some extent the large residual polarization associated with PVTC.

As shown in Figure 4c, at the maximum applied electric field (400 kV/mm), the efficiency of the pristine BOPP film remains at 94%, but the maximum discharge energy density is only 1.9 J/cm^3^. In the case of the pristine PVTC film, when an electric field of 350 kV/mm was applied, the maximum discharge energy density was 7.2 J/cm^3^, with an efficiency of 49%. In the bilayer films, as the volume ratio of BOPP increases, the energy storage density gradually increases; the PVTC/BOPP(33%), PVTC/BOPP(50%), and PVTC/BOPP(67%) bilayer films achieved a maximum energy storage density of 8.2 J/cm^3^, 8.5 J/cm^3^, and 10.1 J/cm^3^ at electric field strengths of 450 kV/mm, 500 kV/mm, and 550 kV/mm, respectively. The energy storage efficiency of all the bilayer films gradually decreases with increasing electric field strength. The charge/discharge efficiency of the three bilayer films at the maximum electric field strength is 60.1%, 69.8%, and 80.9%, respectively. We have compared the discharge energy density and charge/discharge efficiency of PVTC/BOPP bilayer films with different BOPP volume fractions and the same thickness under an electric field intensity of 350 kV/mm; the results are presented in Figure 4d. It can be seen that an increase in BOPP volume fraction is accompanied by a decrease in energy storage density and an increase in charge/discharge efficiency. The PVTC/BOPP bilayer films with thicker PVTC layers have a larger relative dielectric constant, which is beneficial for achieving greater potential shift polarization and a high charging energy density. However, the PVTC/BOPP bilayer films with thicker PVTC layers also have a larger residual polarization, which can cause energy loss and result in a low charge/discharge efficiency. This is consistent with the electrical properties discussed above. Therefore, the thicker the PVTC layer, the greater the residual polarization of the bilayer films is, with a consequent lower charge/discharge efficiency. Overall, the bilayer films combine the advantages of BOPP and PVTC, exhibiting a higher potential shift polarization compared with BOPP alone and a higher charge/discharge efficiency than PVTC.

**Figure 5:**
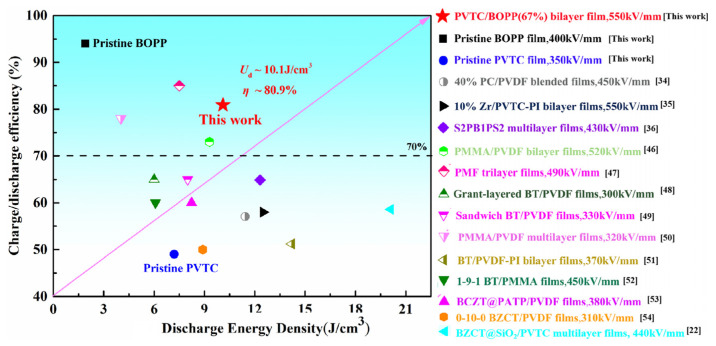
Comparison of the energy density *U*_d_ and charge/discharge efficiency *η* of this work’s PVTC/BOPP(67%) bilayer film and previously reported results [22,34,35,36,46,47,48,49,50,51,52,53,54].

The discharge energy density (*U*_d_) and charge/discharge efficiency (*η*) recorded in this work are compared with recently reported polymer composites [46,47,48,49,50,51,52,53,54] in Figure 5. In all cases, the same matrix (PVDF co-polymer) and testing temperature (room temperature) were used. The majority of the reported *η* values are lower than the efficiency achieved in this study. A low discharge efficiency for a capacitor results in significant Joule heat loss that is detrimental to stable performance. The PVTC/BOPP bilayer films in this work exhibit a good balance of *U*_d_ (10.1 J/cm^3^) and *η* (80.9%) and can effectively address the dilemma that “high *U*_d_ is always accompanied by low *η*”.

## 4. Conclusions

This study has thoroughly examined the impact of BOPP composition on the electrical and energy storage characteristics of PVTC/BOPP bilayer films with heterostructures. At a BOPP content of 67 vol%, the PVTC/BOPP bilayer film exhibited an exceptional energy storage capacity (*U*_d_ = ca. 10.1 J/cm^3^, *η* = ca. 80.9% at 550 kV/mm). When compared with the energy storage performance of pristine PVTC at a maximum field strength of 350 kV/mm (*U*_d_ = ca. 7.2 J/cm^3^, *η* = ca. 49.1%), the *U*_d_ is improved by a factor of 1.4, *η* by a factor of 1.7, and *E*_b_ by a factor of 1.6. By utilizing the dielectric properties of linear/ferroelectric layers, the electric field distribution of the composite film can be controlled, achieving a balance between relative dielectric constant and breakdown field strength, enhancing the energy storage density and charge/discharge efficiency. The concept of polymer-based composites with linear/ferroelectric heterostructures offers a new design paradigm for developing high-performance dielectric materials for flexible energy storage applications, and extends our understanding of dielectric breakdown and polarization mechanisms.

## Data Availability

Data are contained within the article.
